# Dexamethasone Treatment Reverses Cognitive Impairment but Increases Brain Oxidative Stress in Rats Submitted to Pneumococcal Meningitis

**DOI:** 10.1155/2011/173035

**Published:** 2011-11-21

**Authors:** Tatiana Barichello, Ana Lucia B. Santos, Cintia Silvestre, Jaqueline S. Generoso, Andreza L. Cipriano, Fabricia Petronilho, Felipe Dal-Pizzol, Clarissa M. Comim, João Quevedo

**Affiliations:** ^1^Laboratory of Experimental Microbiology and National Institute for Translational Medicine (INCT-TM), Postgraduate Program in Health Sciences, Health Sciences Unit, University of Southern Santa Catarina, 88806-000 Criciúma, SC, Brazil; ^2^Laboratory of Pathophysiology and National Institute for Translational Medicine (INCT-TM), Postgraduate Program in Health Sciences, Health Sciences Unit, University of Southern Santa Catarina, 88806-000 Criciúma, SC, Brazil; ^3^Laboratory of Neurosciences and National Institute for Translational Medicine (INCT-TM), Postgraduate Program in Health Sciences, Health Sciences Unit, University of Southern Santa Catarina, 88806-000 Criciúma, SC, Brazil

## Abstract

Pneumococcal meningitis is associated with a significant mortality rate and neurologic sequelae. The animals received either 10 **μ**L of saline or a *S. pneumoniae* suspension and were randomized into different groups: sham: placebo with dexamethasone 0.7 mg/kg/1 day; placebo with dexamethasone 0.2 mg/kg/7 days; meningitis groups: dexamethasone 0.7 mg/kg/1 day and dexamethasone 0.2 mg/kg/7 days. Ten days after induction we evaluated memory and oxidative stress parameters in hippocampus and cortex. In the step-down inhibitory avoidance task, we observed memory impairment in the meningitis group with dexamethasone 0.2 mg/kg/7 days. The lipid peroxidation was increased in hippocampus in the meningitis groups with dexamethasone and in cortex only in the meningitis group with dexamethasone 0.2 mg/kg/7 days. The protein carbonyl was increased in hippocampus in the meningitis groups with dexamethasone and in cortex in the meningitis groups with and without dexamethasone. There was a decrease in the proteins integrity in hippocampus in all groups receiving treatment with dexamethasone and in cortex in all groups with dexamethasone (0.7 mg/kg/1 day). The mitochondrial superoxide was increased in the hippocampus and cortex in the meningitis group with dexamethasone 0.2 mg/kg/7 days. Our findings demonstrate that dexamethasone reverted cognitive impairment but increased brain oxidative stress in hippocampus and cortex in Wistar rats ten days after pneumococcal meningitis induction.

## 1. Introduction

*Streptococcus pneumoniae* causes the worst acute bacterial infection of the central nervous system (CNS) [1]. Pneumococcal meningitis in adults results in cognitive speed loss [2], intracranial complications, brain edema, hydrocephalus, hippocampal apoptosis, and cortical necrosis [3]. Cognitive impairments were found in one-third of patients, and for this reason, large numbers of patients will continue to have complaints attributable to their illness after the acute phase of the disease [4]. The inflammatory host response in the subarachnoid space seems to be associated with unfavorable outcomes, accompanied by intrathecal production of multiple mediators, including the TNF-*α*, IL-1*β* IL-6 [5], and matrix metalloproteinases; in consequence, polymorphonuclear leukocytes are attracted and activated. As a result, large amounts of nitric oxide and reactive oxygen species (ROS) are produced [6]. The brain is particularly vulnerable to ROS, brain cells' membranes are rich in polyunsaturated fatty acids that can be oxidized [7] leading to lipid peroxidation, DNA modification, and cellular dysfunction [8]. Furthermore, treatments with antioxidants have demonstrated to prevent the brain damage [9], attenuating meningeal inflammation, blood-brain barrier breakdown, and intracranial hypertension [10]. Dexamethasone is a glucocorticoid, very well known to reduce inflammatory cascades, and is used in variety of clinical conditions where their immunosuppressive properties are beneficial [4]. However, the use of dexamethasone in bacterial meningitis is controversial [11]. In experimental animal model with pneumococcal meningitis, dexamethasone inhibited the matrix metalloproteinase expression [12], decreased neurological sequelae, and inhibited caspase-3 activity in adult rat model by Group B Streptococcus [13, 14]. On the other hand, dexamethasone as an adjuvant therapy aggravated hippocampal apoptosis, reduced learning capacity [15], and did not prevent sensorineural hearing loss in pneumococcal meningitis in infant rats [16]. Thereby, in this study, we investigated the adjuvant therapy with dexamethasone in cognitive performance and oxidative stress in rats induced by pneumococcal meningitis.

## 2. Material and Methods

Male Wistar rats (3-4 months, 220–310 g) were obtained from our breeding colony (UNESC). The animals were housed five in a cage with food and water available ad libitum and maintained on a 12 h light/dark cycle (lights on at 7:00 a.m.). All experimental procedures involving animals were performed in accordance with the NIH Guide for the Care and Use of Laboratory Animals and the Brazilian Society for Neuroscience and Behavior (SBNeC) recommendations for animal care and approved by the Animal Care and Experimentation Committee of UNESC, Brazil by protocol 84/2009.

### 2.1. Meningitis Model

All surgical procedures and bacteria administrations were performed under anesthesia consisting of an intraperitoneal (i.p.) administration of ketamine (6.6 mg/kg), xylazine (0.3 mg/kg), and acepromazine (0.16 mg/kg) [17]. *S. pneumoniae* was cultured overnight in 10 mL Todd Hewitt broth medium and grown for another 6 h (35°C, CO_2_ 5.5%) and grown to logarithmic phase. The culture was centrifuged for 10 min at 5,000 × g and resuspended in sterile saline to the desired concentration and used for intracisternal injection [18, 19] containing 5 × 10^9^ cfu/mL [14, 20]. On day 1, the rats underwent a basilar cistern tap with a 23-gauge needle. The position of the needle was verified by the free flow of clear cerebrospinal fluid. Cerebrospinal fluid was withdrawn and the animals received either 10 *μ*L of sterile saline 0.85% as a control (sham) or an equivalent volume of the *S. pneumoniae*. Cerebrospinal fluid (CSF) was obtained by intracisternal puncture at 16 h after infection, and 5 mL were cultured in serial dilutions on blood-agar plates to assess bacterial load [18, 21], followed by the initiation of the antibiotic treatment (ceftriaxone 100 mg/Kg twice a day, i.p., for seven days) [20]. The animals were randomized into different groups: sham: placebo group with dexamethasone (0.7 mg/kg/1 day) and placebo group with dexamethasone (0.2 mg/kg/7 days) and meningitis group with dexamethasone (0.7 mg/kg/1 day) and meningitis group with dexamethasone (0.2 mg/kg/7 days) [22]. Ten days after the induction of meningitis behavioral test was performed. After the behavioral test, the animals were sacrificed, and hippocampus and cortex were isolated to the determination of thiobarbituric acid reactive substances, protein carbonyls groups, sulphydryl groups, and mitochondrial superoxide.

### 2.2. Behavioral Tests

#### 2.2.1. Step-Down Inhibitory Avoidance Task

This task evaluates aversive memory. The apparatus and procedures have been described in previous reports [23]. Briefly, the training apparatus was a 50 × 25 × 25 cm acrylic box (Albarsch, Porto Alegre, Brazil) whose floor consisted of parallel caliber stainless steel bars (1 mm diameter) spaced 1 cm apart. A 7 cm-wide, 2.5 cm-high platform was placed on the floor of the box against the left wall. In the training trial, animals were placed on the platform, and their latency to step down on the grid with all four paws was measured with an automatic device. Immediately after stepping down on the grid, the animals received a 0.4 mA, 2.0 s foot shock and returned to their home cage. A retention test trial was performed 24 h after training (long-term memory). The retention test trial was procedurally identical to training, except that no foot shock was presented. The retention test step-down latency (maximum 180 s) was used as a measure of inhibitory avoidance retention.

### 2.3. Biochemical Assays

#### 2.3.1. Lipid Peroxidation

Lipid peroxidation was measured by formation of thiobarbituric acid (TBA) reactive substances (TBARS) after the method of Esterbauer and Cheeseman [24]. After brain dissection, brain structures were washed with PBS, harvested, and lysed. TBA 0.67% was added to each tube and vortexed. The optical density of each solution was measured in a spectrophotometer at 535 nm. Data were expressed as nmoL of TBARS equivalents per mg of protein.

#### 2.3.2. Protein Carbonyl Formation

Protein carbonyl content was measured in brain homogenates using 2,4-dinitrophenylhydrazine (DNPH) in a spectrophotometric assay [25]. Absorbance was recorded in a spectrophotometer at 370 nm for both DNPH-treated and HCl-treated samples. Protein carbonyl levels were expressed as nmol of carbonyl per mg of protein. All the results were normalized by protein concentration measured by the Lowry assay [26].

#### 2.3.3. Sulphydryl Groups

Total thiol content (Sulphydryl groups –SH) in the brain was determined using the 5,5-dithiobis(2-nitrobenzoic acid) (2-nitrobenzoic acid) method (DTNB). The conditions of DTNB measurement were as described previously [27], with some modifications. The absorbance at 412 nm was measured and amounts of TNB formed (equivalent to the amount of sulphydryl (SH) groups) were calculated. All the results were normalized by protein concentration measured by the Lowry assay [26].

#### 2.3.4. Mitochondrial Superoxide

As an index of electron transporter chain (ETC) uncoupling, the generation of mitochondrial superoxide (O_2_^−^) was determined as previously described [28]. Superoxide dismutase (E.C. 1.15.1.1.) was used at 0.1–0.3 lM final concentration as a negative control to confirm assay specificity.

#### 2.3.5. Statistical Analysis

Data from the inhibitory avoidance task are reported as median and interquartile ranges and comparisons among groups were performed using Mann-Whitney *U* tests. The within individual groups were analyzed by Wilcoxon tests. Data from the biochemical analyses were analyzed by one-way analysis of variance (ANOVA) followed by the Tukey test when F was significant and are expressed as mean ± standard deviation. All analyses were performed using the Statistical Package for the Social Science (version 17.0) software.

## 3. Results

**Figure 1:**
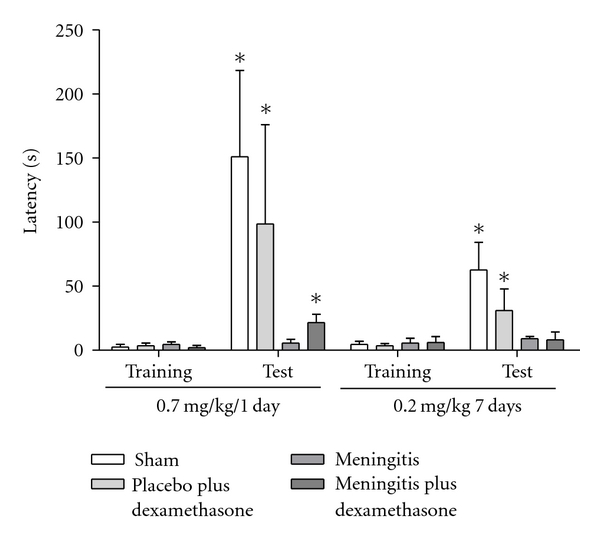
*The step-down inhibitory avoidance. *The step-down inhibitory avoidance test showed meningitis group with dexamethasone treatment (0.7 mg/kg/1 day) and demonstrated group meningitis with dexamethasone treatment (0.2 mg/kg/7 days). Data was presented as media and interquartile ranges, *n* = 10 rats per group. *P < 0.05 versus training.

**Figure 2:**
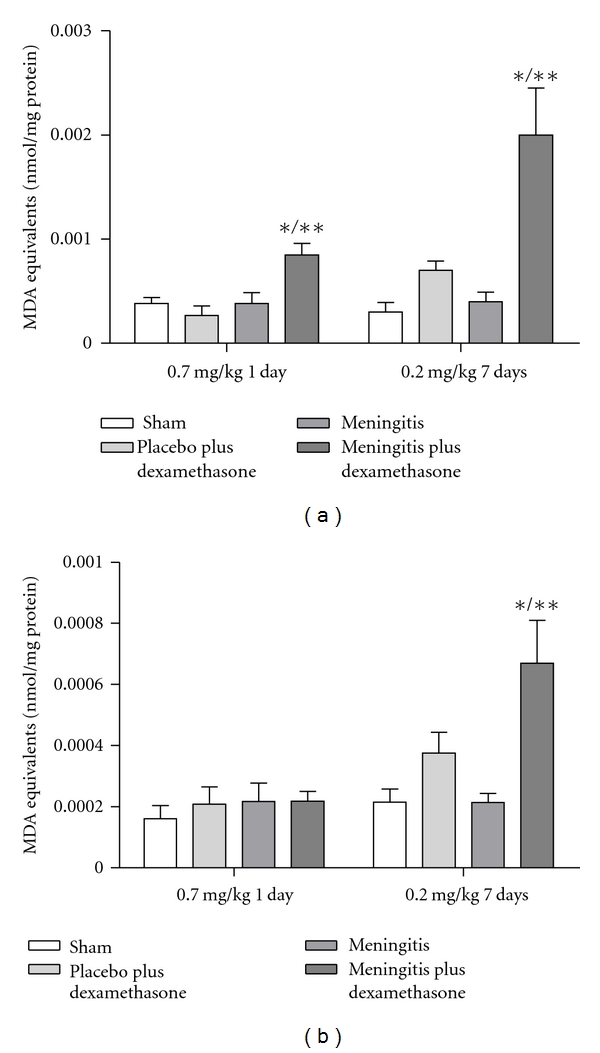
*Oxidative variables. *TBARS. The thiobarbituric acid reactive species (TBARS) was evaluated in the hippocampus (a) and cortex (b), during the treatment with dexamethasone (0.7 mg/kg/1 day or 0.2 mg/kg/7 days). Values are expressed as mean ± SD (*n* = 5 for each group). *Different from sham (*P* < 0.05), **different from sham with dexamethasone.

**Figure 3:**
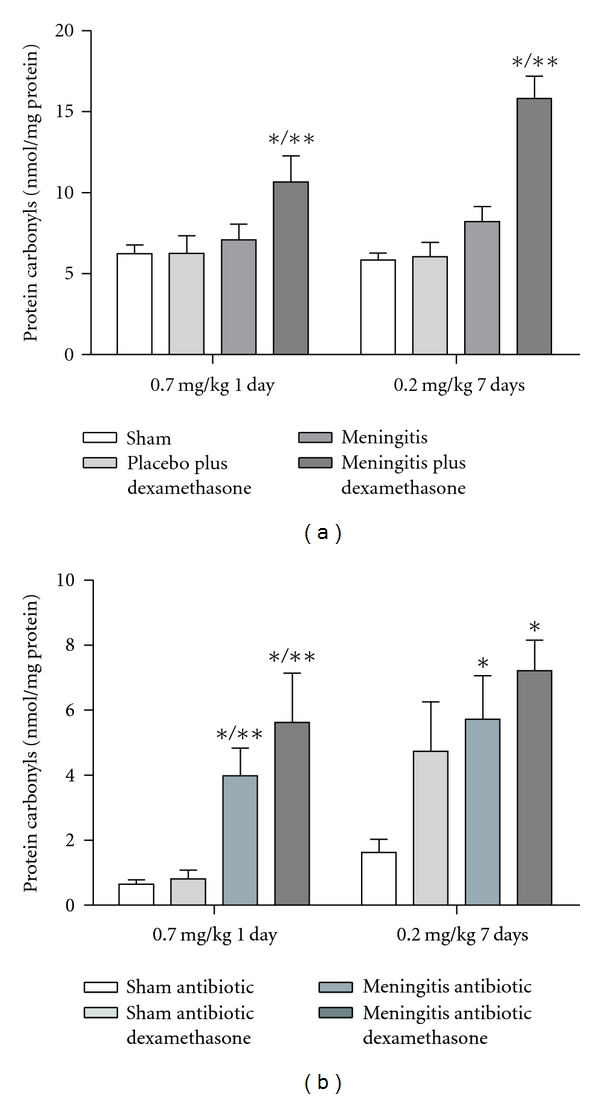
*Protein carbonyl*. The protein carbonyls was evaluated in the hippocampus (a), and cortex (b), during the treatment with dexamethasone (0.7 mg/kg/1 day or 0.2 mg/kg/7 days). Values are expressed as mean ± SD (*n* = 5 for each group). *Different from sham (*P* < 0.05), **different from sham with dexamethasone.

**Figure 4:**
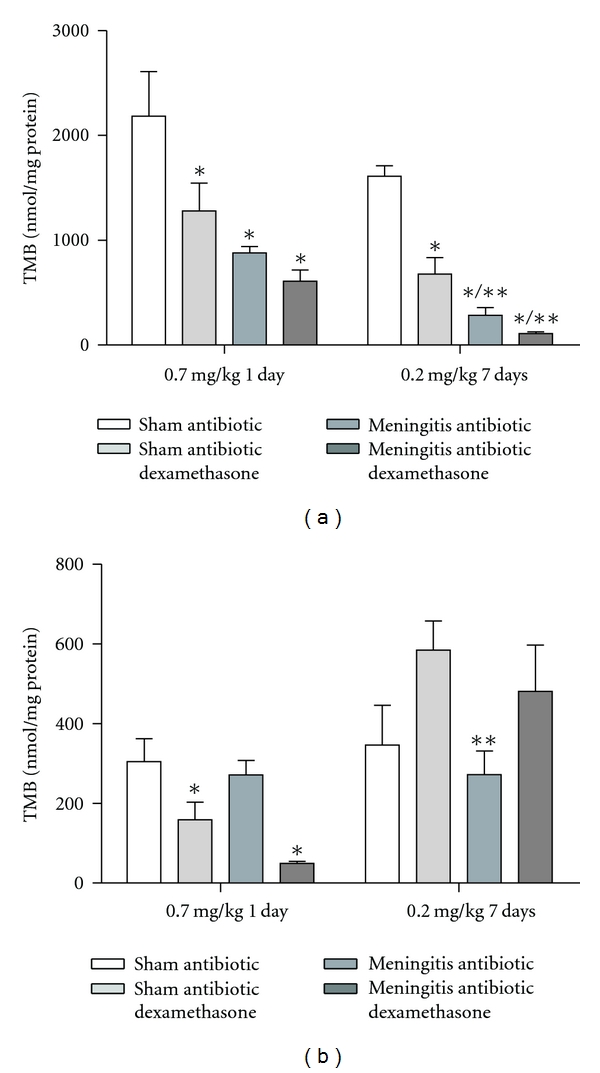
*Sulphydryl groups*. The sulphydryl groups was evaluated in the hippocampus (a) and cortex (b), during the treatment with dexamethasone (0.7 mg/kg/1 day or 0.2 mg/kg/7 days). Values are expressed as mean ± SD (*n* = 5 for each group). *Different from sham (*P* < 0.05), **different from sham with dexamethasone.

**Figure 5:**
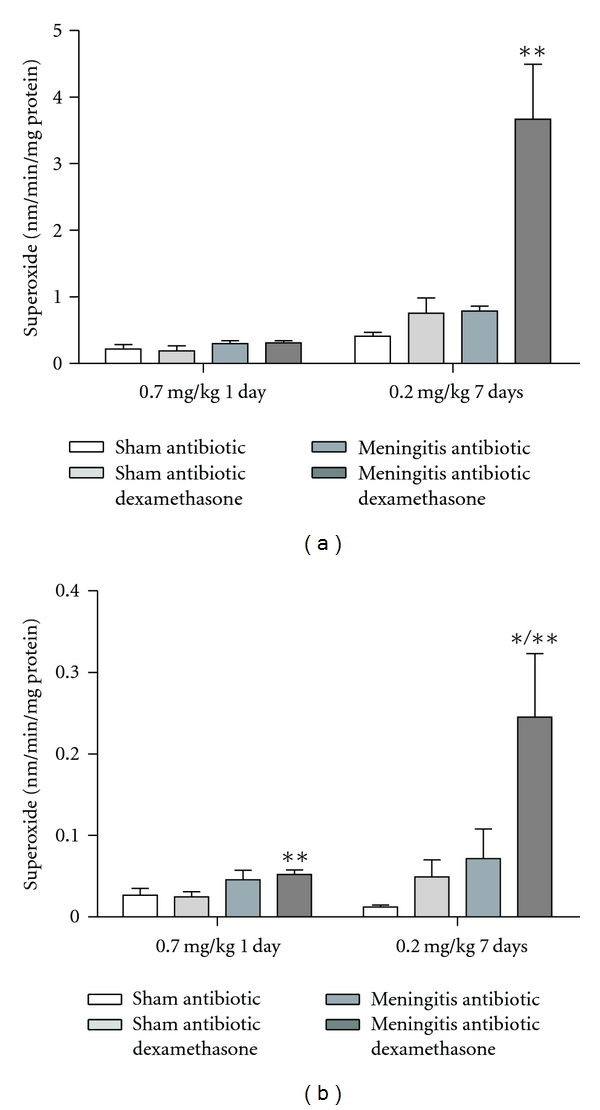
*Mitochondrial superoxide dismutase (SOD)*. The SOD activity was evaluated in the hippocampus (a) and cortex (b) during the treatment with dexamethasone (0.7 mg/kg/1 day or 0.2 mg/kg/7 days). Values are expressed as mean ± SD (*n* = 5 for each group). *Different from sham (*P* < 0.05), **different from sham with dexamethasone.

In Figure 1, we showed results from the step-down inhibitory avoidance test 10 days after pneumococcal meningitis induction. In the acute protocol there were not differences between groups in the training session performance (*P* > 0.05). In the test session, there were not difference between training and test in the step-down latencies in the meningitis group (*P* = 0.153). However, the meningitis group with dexamethasone treatment 0.7 mg/kg/1 day showed statistical difference between training and test sessions (*P* ≤ 0.05). In the repeated dexamethasone protocol, there were not differences between groups in the training session (*P* > 0.05). In the test session, there were not difference in the step-down latency in the meningitis group (*P* = 0.080) and meningitis group treated with dexamethasone 0.2 mg/kg/7 days (*P* = 0.240) when compared to the training session. The oxidative damage in lipids, TBARS, in hippocampus, Figure 2(a), was increased in meningitis groups with dexamethasone (0.7 mg/kg/1 day and 0.2 mg/kg/7 days) when compared with the sham group (*P* ≤ 0.05) and Figure 2(b), in cortex, was increased in meningitis group with dexamethasone treatment (0.2 mg/kg/7 days) when compared with the sham (*P* ≤ 0.05). In Figure 3(a), in hippocampus, protein carbonyl assays, was increased in meningitis group with adjuvant dexamethasone treatment, respectively (0.7 mg/kg/1 day and 0.2 mg/kg/7 days) when compared with the sham group (*P* ≤ 0.05). In Figure 3(b), in cortex, carbonyl levels were increased in meningitis group and meningitis group with dexamethasone treatments, respectively, with (0.7 mg/kg/1 day and 0.2 mg/kg/ days) when compared with the sham group (*P* ≤ 0.05). The integrity of proteins was measured by sulfhydryl (SH) groups in Figure 4(a); in the hippocampus, there was a decrease in the integrity of proteins in all groups with adjuvant treatment with dexamethasone and meningitis group. In cortex, all the groups with dexamethasone treatment (0.7 mg/kg/1 day) were decreased in the protein integrity Figure 4(b) (*P* ≤ 0.05). We demonstrated in Figure 5(a), in hippocampus, and Figure 5(b), in cortex, that the increased activity of mitochondrial superoxide in the meningitis group with dexamethasone treatment (0.2 mg/kg/7 days), when compared with sham group (*P* ≤ 0.05). 

## 4. Discussion

With *S. pneumoniae* proliferation in the subarachnoid space large amounts of subcapsular bacterial components are released, which includes lipopolysaccharide, lipoteichoic acid, pneumolysin, and bacterial DNA [29]. Bacterial components are recognized by Toll-like receptors (TLRs) or other pathogen recognition receptors that lead to the activation of NF-kappaB, and it triggers the inflammatory cytokines expression. In consequence, polymorphonuclear leukocytes are attracted and activated; furthermore, the induction of nitric oxide synthase (iNOS) is expressed, producing large amounts of superoxide anion (O_2_^−^) and nitric oxide (NO), leading to the peroxynitrite formation (ONOO^−^). As a result, oxidative stress leads to cytokines and chemokines activation, enhancement of neutrophil activation, lipid peroxidation, DNA single-strand breaks, and mitochondrial damage [30]. Various pathophysiological alterations are induced, such as increased blood brain barrier permeability, brain edema, intracranial hypertension, and thrombosis, which may cause learning deficits, neuropsychiatric impairment, sensory motor deficits, blindness, and deafness [31]. Glucocorticoids are used in clinical conditions, where their immunosuppressive properties are supposed to account for their beneficial effects [4]. Adjunctive treatment with dexamethasone is associated with an inflammatory process reduction in the subarachnoid space [32]; it decreased the caspase-3 activation, inhibited the MMP-9 expression in the rat brains inoculated with *S. pneumoniae *[12], and improved neurologic outcomes in meningitis [13, 14]. We verified that treatment with dexamethasone administered in a single dose prior the antibiotic therapy prevented the cognitive impairment in animals induced with pneumococcal meningitis. At the same time, this adjuvant treatment induced the lipid peroxidation and protein carbonyls, decreased cellular integrity in hippocampus and increased mitochondrial superoxide activity levels. It has been expressed that steroids reduce the blood-brain permeability and thereby the antibiotics penetration into the subarachnoid space [33]. Irazuzta et al., 2005, verified the performance in the water maze task was improved in animals treated with dexamethasone, suggesting that dexamethasone attenuates meningitis memory deficits [13]. Bacteriolytic antibiotics cause the release of bacterial components that augment the host inflammatory response, which, in turn, contributes to the brain injury in bacterial meningitis [21]. This highly proinflammatory response would be inhibited with the use of dexamethasone treatment before the first antibiotic dose. Leib and coworkers [15] showed a significantly impaired learning performance in infected infant rats by pneumococcal meningitis treated with dexamethasone (0.7 mg/kg with an interval of 8 hours from 18 to 34 h) when compared with the control group. We also verified, in our results, that the prolonged dexamethasone use did not prevent cognitive impairment in animal model. In four trials including children with *Haemophilus influenzae* meningitis, dexamethasone reduced the frequency of neurological sequels and sensorineural hearing loss [34, 35]. Likewise, in the European study, it was showed that adjunctive therapy with dexamethasone reduced the unfavorable outcomes rate from 25 to 15% in adults with bacterial meningitis; adjunctive treatment with dexamethasone was given before or with the first antibiotics dose [36]. Dexamethasone inhibits the production of TNF-*α* and IL-1*β*, reduces brain edema, and limits the increase in cerebrospinal fluid lactate and leukocyte concentrations [4]. van de Beek and colleagues, 2010 [37], published the meta-analysis of 2029 patients from five trials. They verified that adjunctive dexamethasone treatment in acute bacterial meningitis therapy did not seem to significantly reduce death or neurological disability. There were no significant treatment effects in any of the prespecified subgroups. The benefit of adjunctive dexamethasone treatment for all or any subgroup of patients with bacterial meningitis thus remained unproven. We verified that treatment with dexamethasone, with one dose, before the beginning of antibiotic therapy reduces cognitive damage in animals induced with pneumococcal meningitis.
